# Constructing the Schizophrenia Recognition Method Employing GLCM Features from Multiple Brain Regions and Machine Learning Techniques

**DOI:** 10.3390/diagnostics13132140

**Published:** 2023-06-22

**Authors:** Şerife Gengeç Benli, Merve Andaç

**Affiliations:** Department of Biomedical Engineering, Faculty of Engineering, Erciyes University, Kayseri 38280, Turkey; mervecolak.1938@gmail.com

**Keywords:** schizophrenia, machine learning, structural MR images

## Abstract

Accurately diagnosing schizophrenia, a complex psychiatric disorder, is crucial for effectively managing the treatment process and methods. Various types of magnetic resonance (MR) images have the potential to serve as biomarkers for schizophrenia. The aim of this study is to numerically analyze differences in the textural characteristics that may occur in the bilateral amygdala, caudate, pallidum, putamen, and thalamus regions of the brain between individuals with schizophrenia and healthy controls via structural MR images. Towards this aim, Gray Level Co-occurence Matrix (GLCM) features obtained from five regions of the right, left, and bilateral brain were classified using machine learning methods. In addition, it was analyzed in which hemisphere these features were more distinctive and which method among Adaboost, Gradient Boost, eXtreme Gradient Boosting, Random Forest, k-Nearest Neighbors, Linear Discriminant Analysis (LDA), and Naive Bayes had higher classification success. When the results were examined, it was demonstrated that the GLCM features of these five regions in the left hemisphere could be classified as having higher performance in schizophrenia compared to healthy individuals. Using the LDA algorithm, classification success was achieved with a 100% AUC, 94.4% accuracy, 92.31% sensitivity, 100% specificity, and an F1 score of 91.9% in healthy and schizophrenic individuals. Thus, it has been revealed that the textural characteristics of the five predetermined regions, instead of the whole brain, are an important indicator in identifying schizophrenia.

## 1. Introduction

Schizophrenia is indeed a severe mental disorder characterized by both positive and negative symptoms and cognitive impairments [1,2]. Research has shown that individuals with schizophrenia often exhibit structural brain alterations, which may contribute to the cognitive and emotional symptoms seen in schizophrenia [3]. Different combinations of symptoms can be seen in some patients compared to others and even at different points in time in the same patient, thus making the disorder complex. Recently, the use of computational methods in the analysis of neuroimaging data obtained through approaches such as electroencephalography, functional, and structural magnetic resonance (MR) imaging has gained popularity in determining the neurobiological nature of psychiatric disorders. Structural MRI studies in schizophrenia have contributed to our understanding of the neural basis of the disorder and have provided valuable insights into the structural changes associated with the symptoms and cognitive deficits observed in individuals with schizophrenia. Here are some key findings of structural MRI studies, such as gray matter abnormalities [4,5,6], verticular enlargement [7], white matter alterations [4,5], cortical thickness and surface area [8,9,10,11], and neurodevelopmental abnormalities [12,13,14].

In their study, Lee and colleagues aimed to evaluate the brain age variation predicted by six algorithms (ordinary least squares regression, ridge regression, least absolute shrinkage and selection operator regression, elastic-net regression, linear support vector regression, and relevance vector regression) using structural MR images obtained from four different centers. The similarity of performance among the algorithms was compared for each sample using correlation analyses and hierarchical clustering [15]. In a study conducted by Avram and colleagues, the gray matter integrity of the basolateral forebrain cholinergic nuclei (BFCN) region, which is cytoarchitectonically defined, was volumetrically evaluated in 72 individuals with schizophrenia and 73 healthy controls matched for age and gender from the Center of Biomedical Research Excellence (COBRE) dataset. It was assumed that BFCN shows low structural gray matter integrity in schizophrenia, and this change is associated with attention deficits in patients [16]. Alkan and colleagues conducted their study on the relationships between cortical thickness in frontotemporal brain regions and cognitive performance in schizophrenia patients, using the COBRE dataset again with 70 individuals with schizophrenia and 72 healthy controls. After pre-processing the images, they compared the cortical thickness of healthy individuals and individuals with schizophrenia. A strong relationship was found between cortical thickness and visual learning and attention performance in schizophrenia [17]. Oh and colleagues aimed to detect schizophrenia in structural MRI datasets using a deep learning algorithm. Data sets obtained from five different centers were included in the study. All Nifti images were manually evaluated by the authors using the MRIcron software. Deep neural networks trained with multi-center structural MRI datasets demonstrated high sensitivity and specificity in identifying schizophrenia and also achieved an accuracy success rate of 71.6% [18]. Cabral and colleagues investigated the effects of clinical and sociodemographic variables on classification by applying multivariate pattern analysis (MVPA) based on both gray matter (GM) volume and functional connectivity measurements in schizophrenia and healthy controls. With structural MR images, they achieved a classification accuracy of 69.7% in the brain classification related to white matter, gray matter, and cerebrospinal fluid, 70.5% in functional connectivity-based classification, and 75% in the combination of structural and functional-based classification [19]. Lei and colleagues designed a study on structural MRI and resting-state functional MRI data collected from a total of 295 individuals with schizophrenia and 452 healthy controls at five research centers. The classification with gray matter and white matter volume features for structural MR image achieved 75.84% accuracy, whereas the classification with amplitude of low-frequency fluctuation (ALFF) and regional homogeneity (ReHo) features for rs-fMRI achieved 82.75% accuracy. The combined usage of these structural and functional features led to an impressive 90.83% accuracy [20]. Lee and colleagues used volume and trace values of 184 brain regions in a dataset consisting of 47 individuals with schizophrenia and 23 healthy controls in their study based on diffusion tensor imaging and structural MRI. Classification metrics were achieved with high success in random forest with 87.6% sensitivity and 95.9% specificity and in SVM with 89.5% sensitivity and 94.5% specificity [2].

Brain regions including the amygdala, caudate, pallidum, putamen, and thalamus have been investigated using various approaches and associated with schizophrenia [21,22]. However, the study proposed here aims to contribute to the literature by addressing the following unique aspects:

- This study focuses on subcortical structures such as the amygdala, caudate, pallidum, putamen, and thalamus. While these regions have been studied in relation to schizophrenia before, this study may offer new insights or findings specific to these regions.
- Unlike the datasets used in previous studies that targeted specific brain regions mentioned in the literature, the COBRE dataset was utilized providing an opportunity to examine the relationship between subcortical structures and schizophrenia using a specific dataset.
- For the first time, feature extraction was performed using the GLCM technique on the subcortical focused brain regions.
- High classification accuracies were achieved using various classification algorithms in three conditions: right hemisphere, left hemisphere, and bilateral hemispheres, based on the GLCM features of these subcortical regions.
- The detectability of hidden patterns in structural MR images belonging to five focused regions outside the whole brain using GLCM features was analyzed.

Thus, this proposed study emphasizes the potential of these selected brain regions and the texture features obtained using GLCM with combination of the machine learning algorithms to reflect the nature of schizophrenia without examining the whole brain.

## 2. Materials and Methods

The dataset, preprocessing, segmentation, feature extraction–selection, and classification methods used for the proposed study are explained in this section in detail.

### 2.1. Dataset

The raw data were obtained as structural MR images from the Mind Research Network Center of Biomedical Research Excellence (COBRE) (http://fcon_1000.projects.nitrc.org/indi/retro/cobre.html) (accessed on 20 September 2020). The COBRE dataset includes 72 individuals with schizophrenia and 75 healthy controls (ages ranging from 18 to 65 in each group). All subjects were screened and discarded if they had a history of mental retardation, a history of neurological disorder, a history of severe head trauma with more than 5 min of loss of consciousness, or a history of substance dependence or abuse within the last 12 months. Diagnostic information was collected using the structured clinical interview used for DSM disorders (SCID). Considering various elimination criteria, fifteen healthy controls and 12 individuals with schizophrenia were removed during preprocessing and postprocessing. Therefore, this study was designed with 60 healthy controls and well-matched 60 individuals with schizophrenia. Clinical and demographic features of the dataset used in this study are presented in Table 1. For the group with schizophrenia, Positive and Negative Syndrome Scale (PANSS) scores are given, as are the age of onset of psychotic symptoms and duration of psychotic illness in years.

### 2.2. Structural MRI Data Acquisition

Participants were scanned using 3T TIM Trio scanner with a 12-channel head coil. A multi-echo MPRAGE (MEMPR) sequence was used with the following parameters: TR/TE/TI = 2530/[1.64, 3.5, 5.36, 7.22, 9.08]/900 ms, flip angle = 7°, FOV = 256 × 256 mm^2^, slab thickness = 176 mm, matrix = 256 × 256 × 176, voxel size = 1 × 1 × 1 mm^3^, number of echos = 5, pixel bandwidth = 650 Hz. Participants underwent structural MR scan during 6 min.

### 2.3. Image Preprocessing and Segmentation

Pre-processing procedures were performed in an attempt to improve image quality by reducing or suppressing undesired image distortion and increasing certain image attributes that are suitable for segmentation. Following that, segmentation was performed to extract the real information data from the input image so that further processing could be performed.

Some pre-processing processes, such as skull stripping and normalization, were necessary for brain tissue segmentation. Skull stripping, as the initial stage in brain tissue segmentation, is critical for the accuracy of the analysis, particularly in structural MRI-based brain tissue investigations like this one. During skull stripping, extracerebral tissues such as the skull, eyes, and skin are removed using the BET-Intracranial segmentation technique. The removal of the skull facilitates image processing techniques such as surface rendering, cortical flattening, image registration, and tissue segmentation. As the first step in FSL segmentation, the “Brain Extraction Tool version 2.1 (BET)” provided in FSL software was used to conduct skull stripping and exclude non-brain portions of the image. The fractional density threshold parameter(f) governs the boundary between brain and non-brain tissue [23]. BET has been demonstrated to eliminate too many brain areas from images when performed on our data with a value of f = 0.5 (the default option). As a result, f = 0.13 was optimized and applied to all images using the bet2 command.

FIRST, FMRIB’s Integrated Registration and Segmentation Tool [24], which is one of the most prominent software tools for automated subcortical segmentation based on T1-weighted images, was used for automatic segmentation. The regions of interest (ROI) in this research were identified to be bilateral amygdala, caudate, pallidum, putamen, and thalamus in both groups of subjects. As a result, before evaluating any group differences, the surfaces must all be aligned to a common space. The ROIs mentioned above were segmented using a two-stage affine registration to standard space. The first step is a 12-DOF registration to the nonlinear MNI152 template, followed by a 12-DOF registration with a subcortical mask to eliminate voxels beyond the subcortical regions. The FIRST module performs segmentation on images by applying mode number and boundary correction with parameters optimized for each structure.

### 2.4. Feature Extraction

Texture analysis is concerned with identifying a distinctive means of capturing the underlying properties of textures and expressing them in a simpler and distinct manner. Gray level co-occurrence matrices (GLCMs) are square matrices where the number of rows and columns is equal to the number of gray levels, N, in the image. Each entry (i, j) in the matrix represents the frequency with which the gray level i occurs adjacent to gray level j in the image. More precisely, the adjacency is defined by a displacement vector d = (dx, dy), which specifies both a distance and direction. Common choices for the direction include horizontal (0°), vertical (90°), and two diagonals (45° and 135°), and the default mode as horizontal direction was used in this study. The relationship of gray intensities between two adjacent pixels of an image [reference pixel (i), neighbor pixel (j)] is measured here to provide information about fluctuations in intensity at a pixel of interest [25,26,27].

GLCM of tissues obtained structural MR image can be identified as a rich source of statistical texture features that can be used in training robust machine learning (ML) models, which is a powerful method that is commonly used to identify unique patterns of texture feature distribution within an image [28]. GLCM texture feature extraction is a statistical approach for revealing certain features about the spatial distribution of gray levels in image texture while taking the spatial connection of pixels into account. The regions of interest (ROI) in this study were identified to be bilateral amygdala, caudate, pallidum, putamen, and thalamus in both of two groups. The color images corresponding to each focused brain region obtained from the segmentation results were converted to grayscale. Several texture properties were computed from these identified regions via GLCM, providing a quantitative description of the image texture. In this study, second-order statistical features such as autocorrelation, cluster prominence, cluster shade, contrast, correlation1, correlation2, difference entropy, difference variance, dissimilarity, energy, entropy, homogenity1, homogenity2, maximum probability, sum average, sum entropy, sum of squares, sum variance, information measure of correlation1, information measure of correlation2, inverse difference, inverse difference normalized, inverse different moment, kurtosis, skewness, maximal correlation coefficient, and mean were calculated using the GLCM for bilateral five focused brain regions. Thus, a total of 27 statistical features were obtained for each brain region. The calculation formulas for these features can also be found in Supplementary Table S1.

### 2.5. Feature Selection

From the GLCM features, a feature set for this study was created. The chi-square approach was used to choose features from this feature collection. The chi-square approach is often used in feature selection to assess the connection between each feature and the target variable in a classification problem. The chi-square statistic is generated for each feature by contrasting the observed class frequencies with the anticipated frequencies under the supposition that there is no link between the feature and the target variable. Higher chi-square values for features are seen to be more discriminative or informative in separating various groups. In order to enhance the performance of classification models through feature selection, the chi-square approach is often employed in classification research [29]. In this study, a total of 270 features were extracted from five brain regions in bilateral hemispheres (2 × 5 × 27 = 270). Additionally, for each hemisphere (right and left), 135 features were extracted (5 × 27 = 135). The applied chi-square method determined that 22 of the total features were the most important ones.

### 2.6. Classification

Following the feature extraction and selection processes described above, several machine learning techniques were applied and compared for their ability to classify healthy and schizophrenia groups. These algorithms included Adaboost, Gradient Boost (Gboost), eXtreme Gradient Boosting (XGboost), Random Forest, k-Nearest Neighbors (kNN), Linear Discriminant Analysis (LDA), and Naive Bayes (NB). The algorithms that were used are briefly explained here.

Adaboost, short for ‘adaptive boosting’ is a classifier that transforms weak learners into strong ones to solve classification problems. AdaBoost creates ‘n’ decision trees during the data training process. These newly formed decision trees continue their process by generating a dataset from data misclassified in the preceding tree. While combining the results, it also calculates how much weight should be given to the classifier’s proposed answer [30].

GBoost is a combination of “gradient descent” and “boosting”. It allows for the optimization of any differentiable loss function and is composed of decision trees built incrementally. In summary, it improves the results of decision trees by using the gradient descent algorithm [31].

XGBoost is an optimized version of the Gradient Boosting algorithm, using various hardware and software optimization techniques to achieve superior results with less resource consumption.

Random Forest is a supervised classifier that generates multiple decision trees and combines them to obtain a more stable and better prediction. RF operates by selecting a random subset from the dataset for each tree it creates. It determines the error and importance of a variable by selecting the best-classified variable in each node, thereby performing the classification process [32].

The LDA is a linear classifier that uses hyperlanes to distinguish data. These hyperlanes maximize the interclass mean while keeping the variance between classes to a minimum [33].

kNN is a simple yet efficient classifier, because it is a typical example-based or memory-based learning scheme. The value of k is determined by the user, and the points closest to the test data are determined using Euclidian distance. These points create the feature space. As the value of k increases, the impact of noise on the classifier decreases, but the boundaries between classes become less distinct [34].

The NB algorithm is a machine learning algorithm that assists in classification by calculating conditional probability values for test data. It applies Bayes’ theorems for computation and uses class levels represented as feature values or prediction vectors for classification. It can be implemented using Gaussian, Multinomial, and Bernoulli distributions. In our classification, it was carried out using a Gaussian distribution [35].

**Figure 1:**
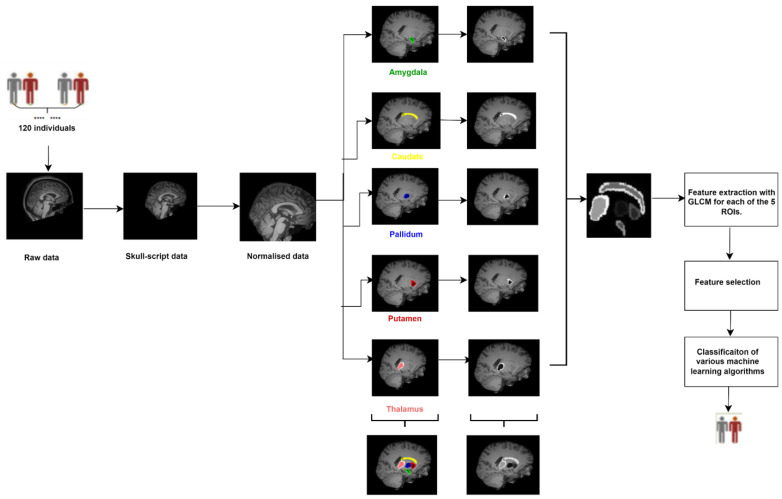
Schematic representation of the proposed classification framework.

All algorithms for our classification process were implemented through Python software using the scikit-learn package. The early stopping function was utilized. The proposed method in this study is summarized in Figure 1, covering the entire process.

## 3. Results

In this study, a method based on the GLCM feature is proposed to distinguish the schizophrenia group from the demographically matched control group. The classification success of combining GLCM features and specific subcortical regions was examined using different approaches, including various boost algorithms, random forests, linear discriminant analysis, k-nearest neighbors, and naive Bayes. The classification performance measures, such as area under the curve (AUC), accuracy, sensitivity, specificity, and F1, obtained from binary classification were evaluated. After randomly shuffling the order of the data through random permutation, machine learning models were built using 70% of the data for training and 30% for testing. The performance parameters obtained from the classification of features extracted from the right brain, left brain, and bilateral brain regions can be seen, respectively, in Table 2, Table 3 and Table 4.

## 4. Discussion and Conclusions

In this study, high-performance classification processes were performed using GLCM features derived from structural MR images of the thalamus, putamen, pallidum, caudate, and amygdala regions in schizophrenia, utilizing machine learning methods.

Upon reviewing the literature, it can be seen that different studies aimed at identifying schizophrenia from MR images have been performed. A literature summary regarding these studies is provided in Table 5. As can be seen from this, research into the classification of schizophrenia has primarily been focused on the whole brain, gray matter, and white matter regions. Moreover, a limited number of machine learning methods have been used. However, in this study, distinct from the studies in the literature, features obtained from five brain regions—right, left, and both hemispheres—have been analyzed. When the classification performances were examined, it was observed that the classification metrics obtained from the left hemisphere performed at a higher level compared to features obtained from the right and bilateral hemispheres. For example, when using the LDA algorithm, 100% AUC, 94.4% accuracy, 92.31% sensitivity, 100% specificity, and an F1 score of 91.9% were achieved. Based on these results, it is observed that various classification studies conducted with GLCM features obtained from the left brain have been more successful compared to the literature studies, shown in Table 5. Even though there are studies in the literature that utilize GLCM features [36,37], the data sets used, the number of data points, classifiers, and the analyzed brain regions show variation in these studies.

The conducted study analyzed in detail the performance of various classifiers and the textural characteristics of the regions we focused on in the right and left hemispheres for the diagnosis of schizophrenia. In future studies, the goal will be to evaluate the performance of GLCM features along with other textural characteristics for the diagnosis of schizophrenia and also to assess them using other datasets.

## Figures and Tables

**Table 1:** Clinical and demographic features.

	Individuals with Schizophrenia (n = 60)	Healthy Controls (n = 60)
Age	34.03	31.87
Gender (Male/Female)	48/12	40/20
Handedness (Right/Left/Both)	(50/9/1)	52/7/1
Age of first episode	20.6	-
Age of onset (Years)	21.1	-
Positive (PANSS)	15.0	-
Negative (PANSS)	14.8	-
General (PANSS)	29.6	-

**Table 2:** Evaluation metrics of various classification results related to the right hemisphere regions.

Right Hemisphere	AUC	Accuracy	Sensitivity	Specificity	F1
Adaboost	82.31	80.43	84.65	80.0	72.70
GBoost	88.29	83.13	71.43	90.19	86.96
XGBoost	79.01	83.34	88.90	76.78	82.35
Random forest	86.25	87.88	75.00	100	90.89
LDA	95.38	88.90	92.31	80.0	80.02
kNN	84.42	83.34	100	72.7	84.2
NB(G)	85.0	77.78	80.0	75.0	75.0

**Table 3:** Evaluation metrics of various classification results related to the left hemisphere regions.

Left Hemisphere	AUC	Accuracy	Sensitivity	Specificity	F1
Adaboost	96.15	91.44	92.31	100	90.3
GBoost	100	94.35	85.71	100	95.65
XGBoost	86.63	83.34	66.57	100	95.65
Random forest	100	89.01	100	80.0	88.78
LDA	100	94.4	92.31	100	91.90
kNN	90.0	88.89	100	80.0	89.04
NB(G)	98.75	83.92	70.0	100	84.21

**Table 4:** Evaluation metrics of various classification results related to the bilateral hemisphere regions.

Right + Left Hemispheres	AUC	Accuracy	Sensitivity	Specificity	F1
Adaboost	96.25	88.98	87.5	90.0	90.0
GBoost	97.14	91.67	93.33	90.47	92.68
XGBoost	97.19	91.67	90.0	93.75	90.91
Random forest	88.57	85.47	86.67	80.95	85.0
LDA	97.84	86.11	94.44	77.78	84.85
kNN	90.0	83.33	93.75	75.0	83.34
NB(G)	90.26	83.74	90.91	71.42	76.92

**Table 5:** Summary of reviewed articles based on feature extraction and machine learning approaches classification of schizophrenia using structural MR images.

Refs.	Dataset	Schizophrenia /Healthy Control (Numbers)	Brain Regions	Extracted Features	Machine Learning Techniques	Accuracy (%)
[38] 2014	Collected data	66/66	Whole brain	Gray matter densities	SVM	90
[39] 2015	Collected data	49/49	Whole brain	Imaging features: deformations MR intensities, Gray matter densities	The modified maximum uncertainty linear discriminant analysis	81.6
[40] 2016	Collected data	41/42	Gray matter and white matter	Gray matter and white matter volumes	SVM	88.4
[41] 2017	Collected data	38/38	Described cortical regions	Cortical thickness features	SVM	88.72
[37] 2018	NAMIC database	20/20	Whole brain	Hu moments, GLCM, Zernika moments, Structure tensor	SVM and Fuzzy SVM	90
[42] 2019	Collected data	40/29	Whole brain	Cortical thickness, gray matter volume, surface area, mean curvature, curvature index and folding index	Multi kernel SVM	71.19
[43] 2020	COBRE dataset	34/34	Gray matter, white matter	Selected gray matter and white matter features	SVM	85.27
[44] 2020	Collected data	50/51 49/48	Whole brain	Voxels of mean gray matter image	SVM	72.2 72.3
[45] 2020	COBRE dataset	57/69	Amygdaloid and hippocampal subregions	Morphological features	SVM	81.75
[46] 2022	Collected data	158/76	Cortical and subcortical brain areas	Cortical and subcortical volume, cortical surface area, cortical mean curvature and cortical thickness	kNN, Logistic regression, SVM, Decision trees, Random forests	Range of 83–87
[36] 2022	Three different data centers	A: 137/132 B: 62/94 C: 144/181	Gray matter, white matter, cerebrospinal fluid, and lateral ventricles	Texture features obtained by GLCM	SVM	A: 66.67 B: 75.00 C: 70.83
[47] 2022	Collected data and B-SNIP dataset	163/173 133/250	Selected some brain regions	Subcortical volumes from seven regions (thalamus, caudate, putamen, pallidum, hippocampus, amygdala, and nucleus accumbens), cortical thickness and cortical surface area measures were extracted for 34 gray matter regions	Significant group difference at *p* < 0.05	Significant differences were obtained between the groups
[48] 2022	NUSDAST dataset, IMH dataset	141/134 148/76	Whole brain	Probability maps of gray matter, white matter, and cerebrospinal fluid	Naive 3D CNN models	79.27 70.98
[49] 2022	Collected data	52/52	Whole brain	(i) Image registration with skull stripping and two automated morphometry methods, (ii) voxel-based morphometry, and (iii) deformation-based morphometry	Autoencoders and 3D-CNN	69.62 62.31
Proposed study	COBRE dataset	60/60	Thalamus, caudate, putamen, pallidum, and amygdala	GLCM features	Adaboost, Gboost, XGboost, Random forest, LDA, kNN, NB	94.4 with LDA from left hemisphere features

## Data Availability

The dataset used is publicly available at the given link below. Once a user account has been created and access is requested, data access will be granted after a short period of time. http://fcon_1000.projects.nitrc.org/indi/retro/cobre.html (accessed on 20 September 2020.)

## Supplementary Materials

The following supporting information can be downloaded at: https://www.mdpi.com/article/10.3390/diagnostics13132140/s1, Table S1: GLCM Texture Features [50,51,52,53].
