# Supplementary material for: Constructing the Schizophrenia Recognition Method Employing GLCM Features from Multiple Brain Regions and Machine Learning Techniques
Source: Diagnostics (Basel). 2023 Jun 22;13(13):2140. doi: 10.3390/diagnostics13132140 (PMC10340621; doi:10.3390/diagnostics13132140)
Supplement: Supplementary file 1 [file diagnostics-13-02140-s001.zip › diagnostics-2453602-supplementary.pdf]

Table S1. GLCM Texture Features

Features	Definitions
Autocorrelation	$\sum_{i=0}^{N}\sum_{j=0}^{N}(i*j)*P(i,j))$
Cluster prominence	$\sum_{i=0}^{N}\sum_{j=0}^{N}(i+j-2\mu)^3*P(i,j))$
Cluster shade	$\sum_{i=0}^{N}\sum_{j=0}^{N}(i+j-2\mu)^4*P(i,j))$
Contrast	$\sum_{\mathrm{i}=0}^{N}\sum_{\mathrm{j}=0}^{N}((\mathrm{i}-\mathrm{j})^2P(i,j))$
Correlation1	$\frac{\sum_{i=0}^{N}\sum_{j=0}^{N}\left((i,j)P(i,j)\right)-M_xM_y}{\sigma_x\sigma_y}$
Correlation2	$\frac{\sum_{i=0}^{N}\sum_{j=0}^{N}(i-\mu_x)(j-\mu_y)P(i,j)}{\sigma_x\sigma_y}$
Difference entropy	$-\sum_{k=0}^{N-1}P_{x-y}(i)\log\left(P_{x-y}(k)\right)$
Difference variance	$\sum_{k=0}^{N-1}\left(k-\mu_{x-y}\right)^2P_{x-y}(k)$
Dissimilarity	$\sum_{i=0}^{N}\sum_{j=0}^{N}\|i-j\|*P(i,j))$
Energy	$\sum_{\mathrm{x}=0}^{N}\sum_{\mathrm{y}=0}^{N}(P^2(\mathrm{i},\mathrm{j}))$
Entropy	$\sum_{i=0}^{N}\sum_{j=0}^{N}p(i,j)\,log(P(i,j))$
Homogeneity1	$\sum_{i=0}^{N}\sum_{j=0}^{N}\frac{1}{1+(i-j)^2}*P(i,j)$
Homogeneity2	$\sum_{i}\sum_{j}\frac{1}{1+\|i-j\|}*P(i,j)$
Maximum probability	$Max_{i,j}P(i,j)$
Sum average	$\sum_{k=0}^{2N-2}k\,P_{x+y}^{(k)}$
Sum entropy	$-\sum_{k=0}^{2N-2}P_{x+y}(k)\log\left(P_{x+y}(k)\right)$
Sum of squares (Varience)	$\sum_{i=0}^{N}\sum_{j=0}^{N}(k-\mu)^2P(i,j)$
Sum variance	$\sum_{k=2}^{2N}(k-\mu_{x+y})^2\,P_{x+y}^{(k)}$

Information measure of correlation1	$\frac{HXY - HXY1}{\text{Max}\,(HX, HY)}$
Information measure of correlation2	$\sqrt{(1 - exp[-2(HXY2 - HXY)])}$
Inverse difference	$\sum_{i=0}^{N}\sum_{j=0}^{N}\frac{P(i,j)}{1+\lvert i-j\rvert}$
Inverse difference normalized (INN)	$\sum_{k=0}^{N_g-1}\frac{P_{i-y}(k)}{1+(\frac{k}{N_g})}$
Inverse difference moment normalized	$\sum_{k=0}^{N_g-1}\frac{P_{i-y}(k)}{1+(\frac{k^2}{N_g^2})}$
Kurtosis	$\frac{1}{m*n}\times\frac{\sum(P(i,j)-M)^4}{SD^4}$
Skewness	$\frac{1}{m*n}\times\frac{\sum(P(i,j)-M)^3}{SD^3}$
Maximal correlation coefficient	$\sum_{k}\frac{P(i,k)P(j,k)}{P_x(i)P_y(j)}$
Mean	$\frac{1}{N_g^2}\sum_{i}\sum_{j}P(i,j)$

1. Soh, L-K, and Costas Tsatsoulis. "Texture Analysis of Sar Sea Ice Imagery Using Gray Level Co-Occurrence Matrices." IEEE Transactions on geoscience and remote sensing 37, no. 2 (1999): 780-95.
2. Haralick, Robert M, Karthikeyan Shanmugam, and Its' Hak Dinstein. "Textural Features for Image Classification." IEEE Transactions on systems, man, and cybernetics, no. 6 (1973): 610-21.
3. Clausi, David A. "An Analysis of Co-Occurrence Texture Statistics as a Function of Grey Level Quantization." Canadian Journal of remote sensing 28, no. 1 (2002): 45-62.
4. Balakumar, B, Raviraj, P, Divya Devi,E. “Brain Tumor Classification Using Machine Learning Algorithms”, Elysium Journal of Engineering Research and Management, (2016): 4 (2).
